# Thermo-mechano-chemical modeling and computation of thermosetting polymers used in post-installed fastening systems in concrete structures

**DOI:** 10.1007/s00161-020-00939-4

**Published:** 2020-11-01

**Authors:** Bilen Emek Abali, Jan Vorel, Roman Wan-Wendner

**Affiliations:** 1 Chair of Continuum Mechanics and Constitutive Theory, Institute of Mechanics, Technische Universität Berlin, Einsteinufer 5, 10587 Berlin, Germany; 2 Christian Doppler Laboratory LiCRoFast, University of Natural Resources and Life Sciences, Peter-Jordan-Straße 82, 1190 Vienna, Austria; 3 Faculty of Civil Engineering, Czech Technical University in Prague, Thákurova 7, 166 29 Prague 6, Czech Republic; 4 Magnel–Vandepitte Laboratory, Department of Structural Engineering and Building Materials, Ghent University, Technologiepark-Zwijnaarde 60, 9052 Ghent, Belgium

**Keywords:** Continuum mechanics, Thermosetting polymers, Thermomechanics, Curing, Finite element method (FEM)

## Abstract

As thermoset polymers find frequent implementation in engineering design, their application in structural engineering is rather limited. One key reason relies on the ongoing curing process in typical applications such as post-installed adhesive anchors, joints by structural elements or surface-mounted laminates glued by adhesive polymers. Mechanochemistry including curing and aging under thermal as well as mechanical loading causes a multiphysics problem to be discussed. For restricting the variety of material models based on empirical observations, we aim at a thermodynamically sound strategy for modeling thermosets. By providing a careful analysis and clearly identifying the assumptions and simplifications, we present the general framework for modeling and computational implementation of thermo-mechano-chemical processes by using open-source codes.

## Introduction

Legal documents require a long service life, especially in structural engineering, no less than 50 years is usual. As a result, experimental procedures are costly and often not feasible. Nonetheless, a few testing techniques and models exist aiming at a performance prediction for that time horizon given the coupled phenomena of post-curing, creep, and fatigue at periodic thermal or mechanical loading. Further complications arise from the high internal moisture of, e.g., concrete or other building materials which are in contact with the thermosets and that give rise to degradation phenomena, such as hydrolytic aging [1–6] playing a significant role in the design-life of the structure. Different phenomena in many length scales [7–11] necessitate incorporation of multiscale approaches. Chemical reaction leading to hardening (hydration) needs to be considered by including dissipative effects [12] also caused by the internal friction during deformation [13, 14]. Mechanical response change related to the loading rate [15, 16] or modeling damage in concrete [17–19] has to be modeled and solved together with the hydration in order to reach accurate prediction of the design-life. Differing bonding mechanisms between structural parts like concrete and steel is an active research area. In the case of a mechanical interlocking, this so-called apparent adhesion [20] based on friction contact provides only a limited bond with respect to a chemical or dispersive adhesion. Amending adhesion is possible by gluing concrete and steel [21–23] that increases also the time to failure [24]. Such a chemical bond is achieved by a special type of polymer, specifically in this work, we concentrate on this adhesive material at the interface [25] between concrete and steel anchor.

Effected by their high stiffness properties, thermosetting polymers are proposed to be used at the interface. They are already available in the market; mostly an epoxy or vinyl ester is used. By mixing two fluid-like components, one is a multifunctional comonomer and the other one is the hardener, the chemical reaction called curing is ignited forming cross-links (chemical bonds) between polymers leading to an increased stiffness. The curing process never stops until it reaches the fully cured state. This process is of paramount importance for correctly characterizing thermosets. Until the so-called gel point, the process is fast and causes a significant shrinkage leading to residual stresses [26], after the gel point we consider the material as a solid—to be precise a fluid with an infinite viscosity—leading to increased stiffness with further cross-linking. It is possible to track the curing process experimentally [27] by introducing a degree of curing, experimental determination of mechanical response [28–30] is an important parameter to calculate the long term response [31]. Mechanical characterization [32] and thermomechanical considerations [33] especially below the gel point [34, 35], or considering aging [36, 37] alongside to the curing have been studied. The effect of the degree of cure on the mechanical response is inevitable. Various models have been used by exploiting numerical multiscale approaches [38–40] or also simple linear models. Multiphysics approaches with adequate numerical implementations are developed as well [41], not only for thermosets using small displacement assumption [42, 43], but also for large displacements [44–46], has been applied in several applications [47–49], where the curing is established in a controlled environment (i.e. heat treatment of composite elements in the autoclave) such that the material characteristics yield the chemically best configuration. In other words, the polymer attains a fully cured state and the curing process stops. However, this is rarely possible in case of thermosetting polymers that find application in the construction industry and especially reinforced concrete structures. Owing to the nature of constructing a structure in an uncontrolled environment under definitely not optimal conditions, the thermoset polymer will typically fail to reach a fully cured state after the installation such that the curing phenomenon continues during the life-time of the structure. Considering the composition altering due to the curing and its effect on the design-life of the system is of paramount importance. In order to model the whole system response accurately, in this work, we start with a thermodynamically consistent modeling of mechanochemistry in thermosetting polymers. We present a computational implementation with the finite element method by using the open-source packages developed under the FEniCS project [50, 51].

## Modeling the curing process

The underlying thermosetting polymer is a resin being hardened due to the chemical reaction, called curing, leading to cross-links between existing polymer chains. Cross-linking affects (often decreases) the volume per mass, an increase in the overall stiffness, and at the same time altering the temperature due to an exothermal reaction. After mixing an agent to the resin, curing starts by using energy from the environment—mostly heat or radiation energy (UV light) is supplied to level off the temperature. Once the curing starts, from the viscous resin state until the so-called gel point, the curing process is fast. At the gel point the viscosity increases drastically so that we may call the material a solid and the curing process slows down; its rate asymptotically reaches zero at the fully cured state. Degree of cure is a difficult variable to measure. We start with a definition of this variable following [52] by using theory of mixtures in a simplified manner. In a unit volume, *V*, masses of resin, curing agent, and cured solid are introduced $m_\text {R}$, $m_\text {A}$, and $m_\text {S}$, respectively. Their values vary in time; but the sum is constant, $m = m_\text {R}+m_\text {A}+m_\text {S}$, throughout the curing process. We introduce mass fractions:

1 $$\begin{aligned} \begin{aligned} Y_\text {R} =&\frac{m_\text {R}}{m} \ , \ \ Y_\text {A} =&\frac{m_\text {A}}{m} \ , \ \ Y_\text {S} =&\frac{m_\text {S}}{m} \ , \end{aligned}\end{aligned}$$

leading to

2 $$\begin{aligned} \begin{aligned} \sum _\alpha Y_\alpha = Y_\text {R} +Y_\text {A} + Y_\text {S} = 1 . \end{aligned}\end{aligned}$$

Now we introduce the degree of cure, $\omega =\omega (t)$, as “measured” in time, giving the mass fraction of the solid

3 $$\begin{aligned} \begin{aligned} Y_\text {S} = \omega \ , \end{aligned}\end{aligned}$$

under the assumption that the initial mass of solid is zero, $Y_\text {S}(t=0)=0$, such that we have

4 $$\begin{aligned} \begin{aligned} Y_\text {R}(t=0) + Y_\text {A}(t=0) = 1 . \end{aligned}\end{aligned}$$

Consider that the ratio of masses, $Y_\text {R}(t=0)/Y_\text {A}(t=0)$, remains the same during the reaction. In other words, same molar mass is used for resin and agent. Now by using $z = Y_\text {R}(t=0)$, we obtain

5 $$\begin{aligned} \begin{aligned} Y_\text {R}(t) =&\,z (1-\omega ) , \\ Y_\text {A}(t) =&\,(1-z)(1-\omega ) , \\ Y_\text {S}(t) =&\,\omega . \end{aligned}\end{aligned}$$

Hence, the whole formulation for calculating the masses of constituents has been subsumed to the degree of cure (conversion degree), $\omega$. For obtaining its value, an evolution equation is developed dependent on the chemical reaction type. Epoxy resin reaction is based on autocatalysis mechanism [53], effected by hydroxy groups formed during catalysis. Hence, the following evolution equation [54] is found to be useful6where amplitudes, $A_\times$, activation energies, $E_\times$, and powers, *m*, *n*, are constant parameters to be determined. Universal gas constant, *R*, is known. Temperature, *T*, is the key variable altering kinetic rates, $k_\times$, and thus curing rate, , significantly. We refer to [55, Table 1] for parameter values of different materials. Obviously,  is used for determining the current degree of cure in each material point. This model fails to incorporate vitrification such that different mechanisms below and above glass transition temperature, $T_g$, are not modeled accurately. Especially curing at low temperatures (below 100 $^\circ$C) leads to incomplete conversion that is of paramount interest in fastening systems, where the hardening occurs in environmental conditions. A possible approach as in [56] characterizes glassy and rubbery states occurring simultaneously. The idea was introduced in [57] based on phenomenological observation combined with the free volume reduction during cure leading to decrease in mobility. Mobility plays a dominant role in the rubbery state as molecules collide and form a network via cross-linking. Beyond a cross-linking density, material vitrifies and in this glassy state the chemical rate is more dominant. By following [58], we use only primary and secondary amines via autocatalysis and impurity catalysis (denoted by *c* in index), collective kinetic rates, $K_1$, $K_{1c}$, involve diffusion controlled mechanism, $K_\text {diff}$, as well as chemically steered mechanism, $K_{1,\text {chem}}$, $K_{1c,\text {chem}}$, as suggested in [59] as follows:7The diffusion rate is modeled by the Williams–Landel–Ferry (WLF) equation based on [60, 61] in the rubbery regime and greater than chemical rate in many orders in magnitude. In the glassy state, diffusion rate is nearly zero regarding chemical rate. This interplay delivers a realistic prediction of conversion degree, $\omega$, at low temperatures, where a so-called partial freezing inhibits to attain $\omega =1$. This model incorporates glass transition temperature, which is often determined by a fit function [62] based on calorimetric measurements. For consistency, we follow [58] and use the relation from [63]

8 $$\begin{aligned} \begin{aligned} T_g = \exp \bigg ( \frac{ (1-\omega )\ln (T_{g,0}) + \Delta C \omega \ln (T_{g,\infty })}{ (1-\omega ) + \Delta C \omega } \Big ) \ , \end{aligned}\end{aligned}$$

with $\Delta C= \Delta c_\infty /\Delta c_0$ as the ratio of the heat capacity change at the glass transition temperature of the fully cured network with $\omega =1$, $T_{g,\infty }$ per the heat capacity change at the glass transition temperature of the monomer with $\omega =0$, $T_{g,0}$. The necessary coefficients for the evolution equations (6), (7) are determined by inverse analysis to data obtained by differential scanning calorimetry, we compile them from the literature as presented in Table 1. By knowing this mass fraction and the current mass density of the mixture, we can extract masses of each constituent by using Eq. (5) with *z* being the mass fraction of resin initially.

**Table 1:** Parameters for the evolution equation of the conversion degree, $\omega$, given in Eq. (6) (upper part) and in Eq. (7) (lower part) compiled from [58]

Parameter	Variable	Value	Unit
Amplitude	$A_1$	$1.7\times 10^{9}$	1/s
Amplitude	$A_2$	$280\times 10^{3}$	1/s
Universal gas constant	*R*	8.314	J/(mol K) $\hat{=}$ G/K
Activation (energy)	$E_1/R$	$12\times 10^{3}$	K
Activation (energy)	$E_2/R$	$7\times 10^{3}$	K
Power constant	*m*	0.6	–
Power constant	*n*	1.2	–
Amplitude	$A_3$	$10^{3.4}$	1/s
Activation (energy)	$E_3$	41.5	kJ/mol $\hat{=}$ kG
Amplitude	$A_4$	$10^{3.6}$	1/s
Activation (energy)	$E_4$	51.8	kJ/mol $\hat{=}$ kG
Amplitude	$k_{T_g}$	$3.8\times 10^{-5}$	1/s
WLF parameter	$C_1$	40	–
WLF parameter	$C_2$	52	K
Ratio	$\Delta C$	0.57	–
Monomer transition temperature	$T_{g,0}$	-10	$^\circ$C
Fully-cured transition temperature	$T_{g,\infty }$	165	$^\circ$C

## Thermodynamical formulation

Under the simplification that deformation is small such that no distinction is necessary between configurations, we use  as the partial time derivative and $_{,i}$ for the space derivative with respect to $X_i$, where space coordinates, $\varvec{X}$, denote material particles composing a material system. Hence, the mass balance is satisfied and the mass density, $\rho$, is a given function in space—for homogeneous materials we set $\rho =\text {const}$ in space, the presented formalism holds true for heterogeneous materials as well. We need to solve balances of momentum and internal energy,9respectively, where $\varvec{u}$ in m is the displacement to be calculated; stress, $\varvec{\sigma }$ in Pa$\hat{=}$N/m$^2$, heat flux, $\varvec{q}$ in W/m$^2$, and specific internal energy (energy per mass), *u* in J/kg, need to be defined; $\varvec{g}$ in N/kg is the gravitational specific force, and *r* in W/kg is the given specific supply term. For the sake of simplicity, we use a linear strain measure:

10 $$\begin{aligned} \begin{aligned} \varepsilon _{ij} = \frac{1}{2} ( u_{i,j} + u_{j,i} ) \ . \end{aligned}\end{aligned}$$

However, we emphasize that the formulation is also suitable for nonlinear measures. Internal energy needs to be defined by exploiting thermodynamics. There are several methodologies; we refer to [64] for such examples based on thermodynamics of irreversible processes and non-equilibrium thermodynamics. Briefly, we introduce the so-called specific Helmholtz free energy:11with the specific entropy, $\eta$, to be defined indicating the reversible part of heat flux and temperature, *T*, to be calculated. Free energy is modeled by assuming that it depends on temperature, *T*, strain, $\varvec{\varepsilon }$, and degree of cure, $\omega$,12We stress that we choose to exclude a dependency on the rate of these variables. Now by inserting the free energy into the balance of internal energy, dividing by *T*, and after rewriting, we obtain13Based on the nonequilibrium thermodynamics, we decompose

14 $$\begin{aligned} \begin{aligned} \eta = \eta ^\text {eq}+ \eta ^\text {neq}\ , \ \ \varvec{\sigma }= \varvec{\sigma }^\text {eq}+ \varvec{\sigma }^\text {neq}\ , \end{aligned}\end{aligned}$$

into equilibrium and nonequilibrium parts and then use the well-known relations for equilibrium parts15For simplicity we assume that rate of temperature and rate of strain fail to alter mechanical response, leading to $\eta ^\text {neq}=0$, $\varvec{\sigma }^\text {neq}=0$. Equation (15)$_1$ is often introduced as the definition of the entropy and Eq. (15)$_2$ is Cattaneo’s theorem in its general form for small displacements, we refer to [65] for its derivation from a Lagrange function. We stress that these relations show that (equilibrium) entropy and (equilibrium) stress depend on the same arguments as the free energy, $\eta =\eta \big ( T, \varvec{\varepsilon }, \omega \big )$ and $\varvec{\sigma }=\varvec{\sigma }\big ( T, \varvec{\varepsilon }, \omega \big )$, which is often called the principle of equipresence [66, §293.$\eta$]. By using these relations, we arrive at the balance of entropy:16with the right-hand side called the entropy production that is zero for reversible, and it is positive for irreversible processes. By using this assertion—the second law of thermodynamics—we obtain Fourier’s law and the restriction17assuring that the entropy production is (zero or) positive as long as $\kappa \ge 0$. The former is well known in thermodynamics. The latter is equivalent to the postulated Karush–Kuhn–Tucker relation originally introduced in plasticity, for a general discussion we refer to [67]. We emphasize that the chosen evolution equation for $\omega$ in Eq. (6) or (7) is positive such that  has to be negative. Heat is inserted into the system in a standard calorimetric measurement, $\updelta Q = c \, \mathrm{d} T$, with specific capacity, *c*. Now by assuming the relation $T \, \mathrm{d} \eta = \updelta Q$, as proposed in [68] and used in all thermodynamical formulations, we obtain18All these relations will be fulfilled by defining the free energy adequately. After inserting Eq. (17)$_1$ into Eq. (13)$_2$ and using $\eta =\eta \big ( T, \varvec{\varepsilon }, q \big )$ as well as Eq. (15), we acquire19This governing equation for the temperature is a general formulation under the assumption that the free energy depends “only” on temperature, strain, and degree of cure. By defining the free energy adequately, the governing equation will be closed and then calculated numerically.

## Thermo-mechano-chemistry of thermosets

We aim at calculating the deformation by satisfying20and the temperature by fulfilling21for a hardening thermosetting polymer, whose state is characterized by the degree of cure, $\omega$, modeled by the evolution equation (6) or (7). In order to close these governing equations, we need to model the system by defining a specific free energy, . The whole formulation is reduced to define a correct model for the free energy. In the following, we give a possible model and present its relation to the existing models in the literature.

We emphasize that the free energy may depend on temperature, strain, and degree of cure. The real dependence is possibly determined by experiments; there are no thermodynamical restriction about its form. But we know, based on the numerical stability and also partly on our understanding of energy as well as intuition, the energy needs to be positive definite. In full accordance with Eqs. (18), (17)$_2$, we propose to use the following free energy expression,22where all material parameters, *c*, $\varvec{C}$, $\varvec{\alpha }$, $\varvec{\beta }$, $H_\text {{ref}}$, may depend on the same arguments as energy, namely temperature, strain, and degree of cure. This model is the simplest case for a thermoelastic material; the form is quadratic providing stability. The reference temperature, $T_\text {{ref}}$, is often set as the room temperature under two assumptions: the material possesses no thermal stresses and the coefficient of thermal expansion (CTE), $\varvec{\alpha }$, is measured regarding this temperature. Curing-related shrinkage is given by the material parameter, $\varvec{\beta }$. Heat release because of the exothermic reaction is given by $H_\text {{ref}}$, the total heat (per mass) generated by a fully cured specimen. If the material response is such that *c*, $\varvec{C}$, $\varvec{\alpha }$, $\varvec{\beta }$ are constant in $\varvec{\varepsilon }$ and *T*, in other words we neglect hyperelasticity as well as temperature-related changes in material coefficients, then we acquire the (generalized) Hooke’s law with a linear shrinkage and well-known Duhamel–Neumann extension23Analogously, the specific entropy and its derivative with respect to the strain read24In the general case, we expect to have material parameters depending on the cross-linking. A rather obvious observation is made for stiffness, the material hardens as the cross-linking density increases. Accurate modeling of the relationship between material parameters and degree of cure needs to be achieved by experiments [69] by means of detecting  in the aforementioned general formulation. A possible way of introducing the degree of cure is simply to introduce two phases, solid (hardened) and fluid (non-hardened), creating the stiffness in the following simple rule of mixture: $\varvec{C} = (1-\omega ) \varvec{C}^\text {f}+ \omega \varvec{C}^\text {s}$, $\varvec{\alpha }=(1-\omega )\varvec{\alpha }^\text {f}+ \omega \varvec{\alpha }^\text {s}$, where $\varvec{C}^\text {s}$, $\varvec{\alpha }^\text {s}$ and $\varvec{C}^\text {f}$, $\varvec{\alpha }^\text {f}$ mean the maximum stiffness, maximum CTE reached after full cross-linking; and, initial stiffness, initial CTE of the non-hardened fluid alike material, respectively. Even the specific heat capacity, *c*, may depend on the degree of cure, $\omega$, we direct to [70] for such a study. The simple relation between material parameters and degree of cure—motivated by the mixture theory—has a formal benefit of constructing thermodynamically sound material properties [71, Sect. 4]. Since the parameter for shrinkage, $\varvec{\beta }$, is inherently related to the curing process, the explicit (linear) dependence is established in the free energy definition instead of introducing phase depending coefficients. In this manner, we have25such that we reach from Eq. (21)26leading to27Until this stage, we have used the following assumptions:

- empirical fit as in Eq. (6) for the evolution equation of degree of cure,
- small displacements,
- material parameters’ linear dependence on the degree of cure, $\varvec{C} = (1-\omega ) \varvec{C}^\text {f}+ \omega \varvec{C}^\text {s}$, $\varvec{\alpha }=(1-\omega )\varvec{\alpha }^\text {f}+ \omega \varvec{\alpha }^\text {s}$,
- linear material equations provided by the quadratic free energy definition modeled as in Eq. (22).

Especially for modeling fastening systems in concrete, all assumptions are easily justified. Furthermore, for the sake of a better comprehension, we introduce more simplifications as follows:

1. In the material state before cross-linking, stiffness of the fluid-like or gel-type material vanishes, $\varvec{C}^\text {f}=0$, and partly as a consequence, a reversible thermal expansion is negligible, $\varvec{\alpha }^\text {f}=0$. Then Eq. (27) for the temperature is obtained as follows: 28
2. A further simplification, especially beyond the gel point, relies on the fact that the shrinkage and thermal expansion are small. By neglecting these effects, we obtain 29 Especially by neglecting deformation, $\varvec{\varepsilon }=0$, we end up with the formulation used in the literature often for thermal analysis during hardening.
3. Further assumptions emerge in calorimetric measurements, without a supply term, $r=0$. We write the global form of the latter, after using Gauss–Ostrogradskiy theorem and Fourier’s law, 30 The surface normal, $n_i$, is outward the body such that that the second term is the heat released from the body. In an experiment, the heat supplied to the body is measured,
  31 $$\begin{aligned} \begin{aligned} Q = -\int _{\partial {\mathcal {B}}} q_i n_i \, \mathrm{d} A \ . \end{aligned}\end{aligned}$$
  In the case of an experimental setup, where the specimen is almost free on all sides, we may assume that no energy is stored. Furthermore, as the specimen size is small, we assume constant temperature within the body as well as constant rate of curing degree. Heat flow, *Q*, and temperature, *T*, are controlled and measured in the experiment for the specimen of mass, $m = \int _{\mathcal {B}}\rho \, \mathrm{d} V$. For a known specific heat capacity—even if the heat capacity or *H* depends on the temperature, we stress that the temperature is constant within the body, ${\mathcal {B}}$—we calculate 32 The mass, *m*, and mass density, $\rho$, are constant, and we obtain $H_\text {{ref}}$ and parameters in the evolution equation for . Two special cases arise in the measurement. The first case is the isothermal calorimetric measurement, , where the specific heat, *Q*/*m*, is used for determining $H_\text {{ref}}$ and parameters for the evolution equation, . For the former, a full curing state from $\omega =0$ to $\omega =1$ is accomplished and $H_\text {{ref}}$ is calculated. Then, for the latter, a partial curing (even from the same data) is exploited to determine parameters in the chosen evolution equation. The second case is to perform a dynamic test where  is set constant in the equipment in order to characterize the specific heat capacity, *c*.

Various models in the literature are covered in the methodology presented herein. We continue with the general governing equations to demonstrate their predictive capability by computing academic examples.

## Generating the weak form for the finite element method

We consider a general case and set the objective to compute the primitive variables, $\omega$, $\varvec{u}$, *T*, in space, $\varvec{x}$, and time, *t*. By starting with the initial conditions of a partly cured body given by $\omega _\text {{ref}}$ as follows:

33 $$\begin{aligned} \begin{aligned} \omega =\omega _\text {{ref}}\ , \ \ \varvec{u}=0 \ , \ \ T=T_\text {{ref}}\qquad \forall \varvec{x} \in {\mathcal {B}}\ , \ \ t=0 \ , \end{aligned}\end{aligned}$$

within the continuum body ${\mathcal {B}}$, we search for the transient solution of $\{\varvec{u}, T\}$, by solving Eqs. (20), (21) and determine $\omega$ by updating34with the evolution equation for . We use the quadratic free energy as in Eq. (22), leading to linear material equations; yet emphasize that the implementation is designed in such a way that more advanced models are possible to be employed as well. Therefore, we leave the free energy as  in the following. All continuous functions are approximated by using their discrete representations in space and time. In order to discretize in time, we use constant time steps and compute in a time series

35 $$\begin{aligned} \begin{aligned} t = \{ 0, \Delta t, 2\Delta t, 3\Delta t, \dots \} \end{aligned}\end{aligned}$$

with $(\cdot )^0$, $(\cdot )^{00}$ denoting the calculated values of one, two time steps before, respectively. Time derivative is approximated by the finite difference method (Euler backwards scheme)

36 $$\begin{aligned} \begin{aligned} \frac{\partial (\cdot )}{\partial t} = \frac{(\cdot ) - (\cdot )^0}{\Delta t} . \end{aligned}\end{aligned}$$

The governing equations become37We remark the linearized strain measure in order to find out the value one time step before,

38 $$\begin{aligned} \begin{aligned} \varepsilon _{ij} = \frac{1}{2} ( u_{i,j} + u_{j,i} ) \ , \ \ \varepsilon ^0_{ij} = \frac{1}{2} ( u^0_{i,j} + u^0_{j,i} ) \ . \end{aligned}\end{aligned}$$

For space discretization, we use the finite element method with a triangulation of the continuum space, ${\mathcal {B}}$, and exchanging continuous primitive functions with their counterparts in a Hilbertian Sobolev space [72] by using *n*th polynomial degree form functions for each discrete element,

39 $$\begin{aligned} \begin{aligned} \mathcal V = \bigg \{ \{\varvec{u}, T\} \in [\mathcal H^n({\mathcal {B}})]^4 : \{\varvec{u}, T\}\Big |_{\partial {\mathcal {B}}}=\text {given} \bigg \} \ . \end{aligned}\end{aligned}$$

For the sake of notational easiness, we simply skip to distinguish between continuous functions and their discrete representations. We use the standard procedure to construct the integral forms by building residuals from governing equations and multiplying (weighting) them by corresponding test functions, $\updelta \varvec{u}$, $\updelta T$, where they are expressed in the same space as the primitive variables, called the Galerkin procedure,40We emphasize that stress already possesses space derivative of primitive variables, because of the additional divergence on it, we need to choose at least $n=2$ in the space discretization. This continuity condition is weakened by integrating by parts for all second derivative terms,41where we have inserted $\hat{t}_i = n_j \sigma _{ji} = n_j \partial \mathscr {f}/\partial \varepsilon _{ij}$ and $\hat{q} = n_i q_i = -n_i \kappa T_{,i}$ on boundaries. For the displacement with a given traction, $\hat{\varvec{t}}$ in Pa, two sets of boundaries appear: Dirichlet boundaries, $\partial {\mathcal {B}}_D$, and Neumann boundaries, $\partial {\mathcal {B}}_N$. We restrict the formulation such that these two sets fail to intersect, $\partial {\mathcal {B}}_D\cap \partial {\mathcal {B}}_N=\{\}$, so the whole boundary is the union of them, $\partial {\mathcal {B}}=\partial {\mathcal {B}}_D\cup \partial {\mathcal {B}}_N$. At the Dirichlet boundary, the displacement itself is given, hence, no test functions are necessary, $\updelta \varvec{u}=0 \ \forall \varvec{x}\in \partial {\mathcal {B}}_D$. For the rest, traction is given, $\hat{\varvec{t}} =\text {given} \ \forall \varvec{x} \in \partial {\mathcal {B}}_N$. Analogously, the temperature may be modeled by given temperature or heat flux; however, we use a more natural approach by using a mixed boundary condition called Robin boundaries, herein applied to the whole boundary,

42 $$\begin{aligned} \begin{aligned} \hat{q} = h(T-T_\text {a}) \ \forall \varvec{x} \in \partial {\mathcal {B}}\ , \end{aligned}\end{aligned}$$

with the convection parameter, *h*, and the ambient (environment) temperature we use as $T_\text {a}=T_\text {{ref}}$. Two weak forms are in different units, the former is in the unit of energy and the latter is in the unit of power. After multiplying by the constant time step $\Delta t$,43we can sum them up

44 $$\begin{aligned} \begin{aligned} \text {Form} = \text {F}_{\varvec{u}} + \text {F}_{T} \end{aligned}\end{aligned}$$

and use for computing thermomechanics of a curing thermoset.

## Computational implementation and numerical examples

In order to examine the strength and accuracy in predicting the behavior of realistic systems, we implement the weak form in Eq. (44) by using open source packages developed within the FEniCS project. The formulation is implemented in Python, wrapped into C++, compiled, and solved by mpirun in parallel in a Linux computer (Ubuntu 18.04). This scalable implementation is suitable for larger systems as well. Herein we demonstrate simple examples to comprehend the multiphysics in thermo-mechano-chemical systems. Especially two important features need to be emphasized leading to the implementation presented herein.

First, the weak form is nonlinear such that we use the conventional Newton–Raphson linearization approach for solving a linear problem and update the solution by starting from the last converged solution. Even if the system is governed by stiff differential equations, using small time steps leads to a convergence in each iteration. The linearization is handled by exploiting symbolic differentiation that allows to implement also highly nonlinear forms—for example a hyperelastic material model can be easily implemented with the approach developed herein, we refer to [64] as well as to the supply code of [65] for examples of such an implementation.

Second, the specific free energy in Eq. (22) has been implemented also with the aid of symbolic differentiation. Technically, the whole material formulation is reduced to the free energy definition such that the implementation is very general and can be easily adapted for other systems simply by changing the free energy. We stress that the material modeling is limited by the assumed free energy formulation; however, implementation is novel in the sense that any more advanced formulations are possible to utilize. We make the code publicly available in [73] for encouraging its use under GNU Public licensing [74] for an efficient and transparent scientific exchange.

### Curing models comparison

Two evolution equations (6), (7) have been introduced and implemented. Both equations consist of parameters depending on the current temperature such that the weak form $\text {F}_T$ in Eq. (43) is nonlinear. Material parameters belong to typical epoxy type of materials with coefficients compiled in Tables 1, 3. We compute temperature and degree of cure for a simple geometry of 50 mm$\times$10 mm$\times$10 mm, made of homogeneous material. First, we solve the temperature distribution by using model A in Eq. (6), and secondly, we obtain the temperature by applying model B in Eq. (7). Three-dimensional simulation results are recorded, and both results at the same location (geometric midpoint of the domain) are compared qualitatively. We emphasize that the material parameters in Table 1 are realistic; but from different sources such that models A and B fail to be compared quantitatively.

**Fig. 1:**
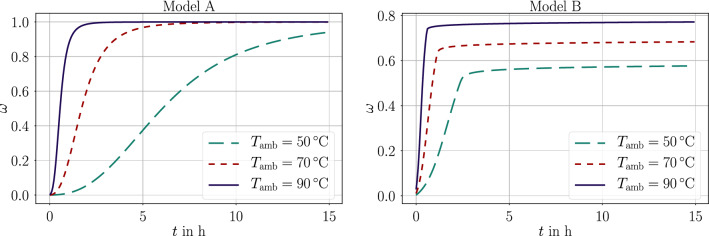
Degree of cure, $\omega$, over time, *t*, under different ambient temperatures computed by the model A (left) as in Eq. (6) and model B (right) as in Eq. (7)

We begin with the well-known effect of the ambient temperature on the curing phenomenon. By setting the convection parameter high, $h=1$ kW/(m$^2$ K), we enforce nearly a constant surface temperature equivalent to the ambient temperature, $T_\text {amb}$. Applying model A and model B leads to results in Fig. 1. As expected, higher temperatures increase the conversion rate such that the possible upper limit is attained in less than 90 minutes at 90 $^\circ$C than in 20 hours at 50 $^\circ$C. These simulations model an isotherm calorimetry measurement. Model A is capable of quantifying the process only in the case of complete curing. We point out the case of an incomplete curing taking place at low temperatures. The model B given by the evolution equation (7) is indeed capable of simulating this freezing behavior effected by the vitrification. Such a vitrification, 10–20 $^\circ$C above the curing temperature, is typically observed in isotherm calorimetry measurements. This glass transition temperature, $T_g$, is also detected and measured with the aid of this observation. During cross-linking, the exotherm reaction produces heat which is taken out of the system in order to hold the temperature constant. For simplicity, we use a constant value for the specific heat capacity in simulations. In reality, the change of specific heat capacity between glassy and rubbery states causes an overshoot in this heat energy, which is used to determine the numerical value of $T_g$ in a non-fully cured structure. Material showing a distinct $T_g$ justifies the use of model B. A typical example are the epoxy systems used in post-installed fastening applications subject to low (ambient) temperature curing.

**Fig. 2:**
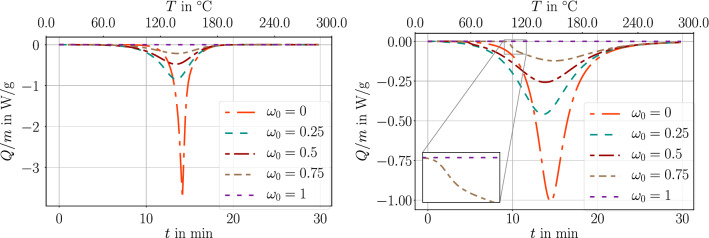
Heat flow in a temperature ramp with two evolution equations: left model A in Eq. (6) and right model B in Eq. (7)

For a better understanding of the role of the evolution equation on the released heat, we simulate a temperature ramp test, where structures with different degrees of cure are simulated as being in a temperature chamber where the ambient temperature increases by 10 K per minute. Figure 2 demonstrates the specific heat flow, *Q*/*m*, obtained by Eq. (32) from the temperature and degree of cure computation. Obviously, model A is symmetric leading to the sigmoid relation, whereas model B shows a kink at the glass transition temperature such that the symmetric character is lost.

In general, the computational implementation results in reliable simulations with realistic parameters. Such a tool is of importance to study and comprehend the behavior of different curing models. We have selected two models without assessment of the quantitative accuracy to a specific material.

### Curing under alternating thermal loading

For a realistic simulation, we use model B and consider that the epoxy with parameters in Table 1 has been used for adhering components in an outdoor facility. Complete curing of the material is desired for an adequate stiffness leading to high adhering. We obtain hourly temperature, as an example on October 1, 2019 in Brussels,^1^ and use with a rough approximation as given in Table 2. By using a convection parameter $h=100$ W/(m$^2$ K), we model a mild windy ambient for heat exchange over the surface. Again for a simple geometry of 50 mm$\times$10 mm$\times$10 mm, we compute transiently the temperature distribution in tree-dimensional continuum coupled to the degree of cure given by the model B. The simulation starts directly after casting, $\omega =0$, computes for 24 hours temperature and degree of cure. The temperature values in the core of the structure are recorded, which are nearly the same as the surface temperature since the structure is small. We emphasize that ambient temperature and structure’s temperature are not identical. Convection over the surface as well as curing produced heat are responsible for this difference.

**Table 2:** Used temperature alternation on one day approximated to the data for Brussels (BE) on October 1, 2019

Time	Temperature in $^\circ$C
12 am	16
2 am	14
9 am	17
12 pm	15
5 pm	20
12 am	15

**Fig. 3:**
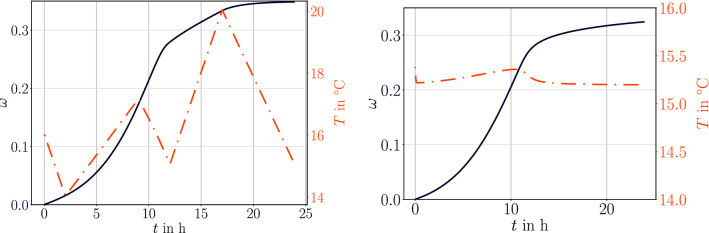
For a comparative analysis, curing of polymer adhesive material as used in Brussels (Belgium) on October 1, 2019. Left: Using the temperature change over the day by using 5 picked values as given in Table 2. Right: Mean temperature value applied on the boundary and shown in the core of the sample

Important observations are attained from results demonstrated in Fig. 3. After one day of curing in environmental temperatures, only $\omega =0.35$ has been reached in Fig. 3(left). With high mobility, rate of conversion is accelerating in the beginning, especially after the early morning when the temperature starts rising. As a coincidence, on this day the temperature drops before noon such that the material presumably vitrifies and afternoon temperature increase is only partly amending the conversion rate. If the material has a gel point of $\omega =0.5$, then 24 hours of curing in a mild autumn day reaches not even beyond the gel point. Obviously, the expected stiffness is not delivered in this condition, at least for this material. Therefore, different commercial products use modified compositions, allowing an increased reaction kinetics in these temperatures. By utilizing the implementation herein, we enable investigating the degree of cure of a material with known parameters. Moreover, we stress that the temperature history is of importance as well. When we use the mean value for the ambient temperature, instead of the alternating temperature, we observe that a significant difference of approximately 10% is captured in Fig. 3(right). Even a linear relation between stiffness and the degree of cure is assumed, this 10% deviation is of importance to consider in design. This analysis is for a small sample, where the temperature conductivity is high enough to generate a homogeneous temperature distribution. However, the exothermal reaction is still altering the temperature in the middle of this sample as visible in Fig. 3(right). But this change is insignificant. Herein, we demonstrate how valuable such a study becomes especially whenever applied to more realistic geometries.

### Mechanical loading superposed by hardening

Under a mechanical loading, the amount of deformation depends on the stiffness that changes during hardening. Two possible implementations can be developed. One approach is to use a stiffness, $\varvec{C}^\text {s}$, for the fluid phase leading to a soft matter even before the gel point. Then we start with $\omega =0$ and with the aid of this initial stiffness, the numerical implementation converges. Another approach is to set the fluid stiffness to zero, $\varvec{C}^\text {s}= 0$, $\varvec{\alpha }^\text {s}= 0$, and start with an initial curing, $\omega _\text {{ref}}$, greater than zero such that the simulation begins with an initial stiffness again leading to a convergence. We choose the second approach, for example the gel point, $\omega _\text {{ref}}=0.5$, where the material response is more solid type than fluid. We use the latter approach in the following simulations. Materials response is chosen to be elastic and the initial geometry is used as the reference configuration to which the continuum body recovers after unloading. This modeling is based on the fact that the hardening beyond $\omega _\text {{ref}}$ fails to affect the reference configuration. We leave an experimental verification of this assumption to further research.

A simple, thick beam of 50 mm$\times$10 mm$\times$10 mm is modeled. Material is assumed to be isotropic such that the solid properties are given by Lame’s constants, $\lambda$, $\mu$, coefficient of thermal expansion, $\alpha$, and shrinkage parameter, $\beta$,

45 $$\begin{aligned} \begin{aligned} C^\text {s}_{ijkl} = \lambda \delta _{ij} \delta _{kl} + \mu \delta _{ik} \delta _{jl} + \mu \delta _{il} \delta _{jk} \ , \ \ \alpha ^\text {s}_{ij} = \alpha \delta _{ij} \ , \ \ \beta _{ij} = \beta \delta _{ij} \ . \end{aligned}\end{aligned}$$

The Lame parameters are related to the (measurable) engineering constants, *E*, $\nu$, as follows:

46 $$\begin{aligned} \begin{aligned} \lambda = \frac{E \nu }{(1+\nu )(1-2\nu )} \ , \ \ \mu = \frac{E}{2(1+\nu )} \ . \end{aligned}\end{aligned}$$

**Fig. 4:**
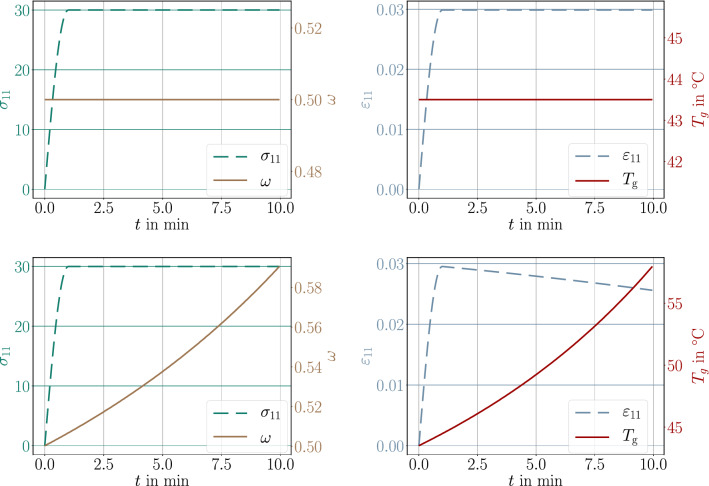
Uniaxial test, (engineering) stress, $\sigma _{11}$, degree of cure, $\omega$, engineering strain, $\varepsilon _{11}$, and glass transition temperature, $T_\text {g}$, for a given ambient temperature. Top: $T_\text {amb}=30\,^\circ$C. Bottom: $T_\text {amb}=60\,^\circ$C

We use fictitious but realistic material coefficients as compiled in Table 3. Nearly all coefficients can be determined by experiments on the corresponding material. But the thermal convection parameter modeling the rate of heat exchange between the surface of the material and environment depends on the material, surface roughness, and ambient state [75]. For example, moving air is known to extract more heat from the material than still air. Analogously, humid air possesses a higher heat transfer coefficient than dry air. We consider a uniaxial tensile test, the beam is clamped on one end and pulled on the other end with a controlled force, first, relatively quickly until it reaches its maximum and then, secondly, it is held at this force throughout the whole simulation of 10 minutes. In the case of an elastic material, the response is instantaneous, the beam gets stretched and remains at the same length under constant force. This case occurs in Fig. 4(top), where the ambient temperature is less than $T_\text {g}$ such that the material remains at the same degree of cure, $\omega _\text {{ref}}$. In the case of an ambient temperature higher than the glass transition temperature, with the additional assumption that Young’s modulus remains the same, response to the applied force is the same; however, the material undergoes further curing. Hence, the material hardens, stiffness increases, and the beam gets shorter under the same force held constantly, see Fig. 4 (bottom). Since we have modeled stiffness to degree of cure linearly, the length change is linear as well. In reality, Young’s modulus depends on the temperature, especially around the glass transition. The resulting decrease of the modulus is significant. Herein we neglect the viscous character and model this behavior by using inverse tangents function and constant moduli for rubbery and glassy states, $E_\text {r}$ and $E_\text {g}$, respectively, as follows:

47 $$\begin{aligned} \begin{aligned} E = \frac{E_\text {g} - E_\text {r}}{2} \Big ( 1 + \frac{2}{\pi }\arctan (T_\text {g} - T) \Big ) + E_\text {r} \ . \end{aligned}\end{aligned}$$

For the simulation, we use again fictitious but realistic parameters, $E_\text {g}=2$ GPa and $E_\text {r}=0.5$ GPa, leading to a stark decrease at the $T_\text {g}$, for example as in Fig. 4. We emphasize that the glass transition temperature, $T_\text {g}$, increases with evolving degree of cure given in Eq. (8). Therefore, we repeat the last analysis and apply a given boundary condition, hold this displacement, increase the ambient temperature, measure the necessary force. In the case of constant Young’s modulus, as we model (linear) elastic material, the response remains the same as long as no postcuring occurs. Since Young’s modulus decreases at the time crossing the current glass transition temperature—we stress that the simulation starts off with $\omega _\text {{ref}}$—stiffness drops because of decreasing Young’s modulus. Furthermore, curing begins and material hardens as well. Such a response is experimentally observed, we refer to [76] for a discussion in this sense. Herein, we observe analogous response in Fig. 6.

**Table 3:** Coefficients and material parameters used in the simulation with SI units

Parameter	Variable	Value	Unit
Mass density	$\rho$	2400	kg/m$^3$
Thermal conductivity	$\kappa$	2.3	J/(s m K)
Specific heat capacity	*c*	1100	J/(kg K)
Coefficient of thermal expansion	$\alpha$	$20\times 10^{-6}$	1/K
Coefficient of shrinkage	$\beta$	$2\times 10^{-5}$	–
Thermal convection parameter	*h*	2–20	J/(s m$^2$ K)
Heat release	$H_\text {{ref}}$	300	J/g
Young’s modulus	*E*	2000	MPa
Poisson’s ratio	$\nu$	0.2	–

**Fig. 5:**
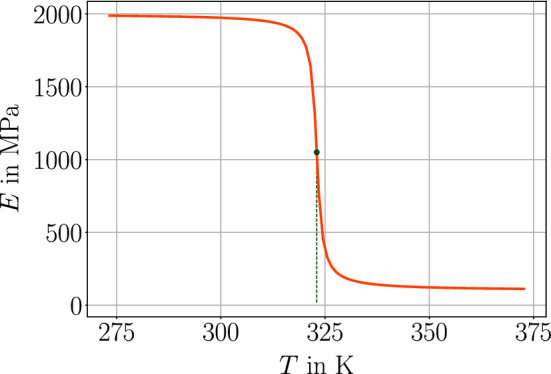
Young’s modulus change during the transition from glassy to the rubbery state, modeled by an arctan function for the case of $T_\text {g}=50^\circ$C

**Fig. 6:**
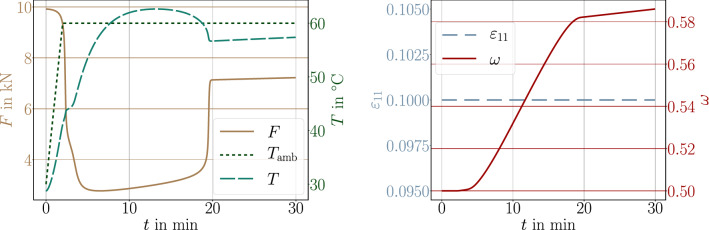
Stress relaxation under constant stretching in three regimes: first, the modulus decreases by crossing to the rubbery state, second, postcuring hardens the material and stiffness increases, third, modulus increases by crossing to the glassy state

**Fig. 7:**
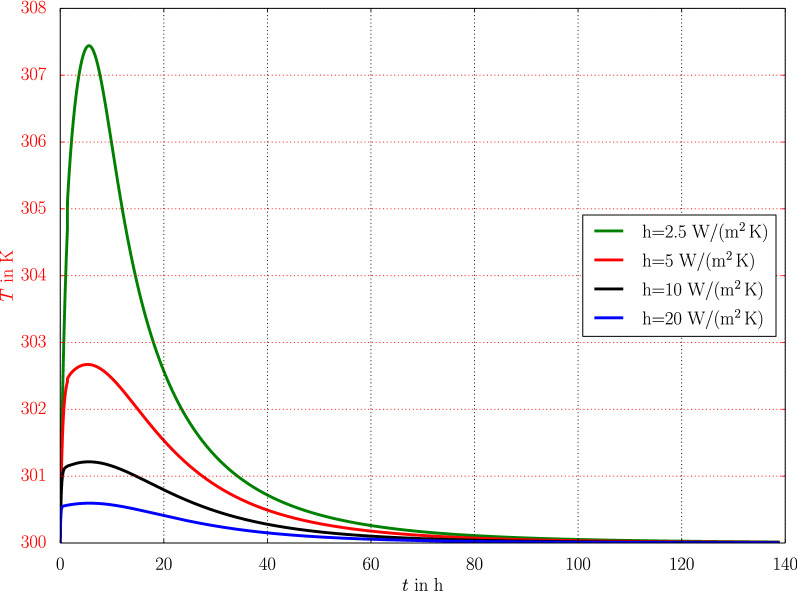
Local temperature evolution for different cases by varying heat transfer coefficient for the same ambient temperature

The curing rate depends on the local temperature that converges to the ambient temperature steered by the convection parameter, *h*. Even for the same ambient temperature $T_\text {{ref}}=300$ K, a variation of the convection parameter shows that the increased heat exchange, larger *h* in the simulation, increases the time necessary for attaining the same degree of cure. This phenomenon is demonstrated in Table 4 quantitatively, where the time is determined, at which, in the middle of the same structure, $\omega =0.95$ has been reached. The justification of this phenomenon is as follows: Increased heat exchange leads to the increasing energy loss to the environment in form of heat energy that is generated as a consequence of the exothermal curing reaction. Obviously, smaller heat transport parameter represents a better isolation from the environment, practically sealing the structure. The generated heat during cross-linking is captured within the material, as seen in the temperature evolution at the middle point of the beam demonstrated in Fig. 7. In other words, the heat energy, which is released free at the moment of curing, increases locally the temperature and accelerates the curing process. The evolution equation for curing depends on the temperature.

**Table 4:** Variation of convection parameter, *h*, and the computed time necessary to attain the state with 95% of cured solid material

*h* in W/(m$^2$ K)	2.5	5	10	20
*t* in min for $\omega =0.95$	51.7	58.6	61.7	63.1

## Conclusion

A general framework has been developed for thermomechanics of thermosetting polymers under curing governed by an evolution equation. The numerical implementation of such a thermo-mechano-chemical process has been established by using open-source packages. Simple geometries are utilized for promoting a better understanding of underlying multiphysics as well as capability of the computational implementation. The following assumptions have been undertaken:

- Small strain formulation is used; there are several applications especially in composite materials, where this assumption is accurate.
- Dissipative parts in the thermal and mechanical terms are neglected. Depending on the chosen material, a viscoelastic term might be necessary.
- The evolution equation for curing depends solely on the temperature. The temperature dependency is intuitive; however, it is not known, if a mechanical deformation would increase the rate of degree of cure.
- The reference configuration is chosen at the gel point, which has been the initial condition as well. Although this assumption is very tempting, we leave an experimental validation of this assumption to further research.

Thermodynamically sound modeling of thermo-mechano-chemical processes allows us to accurately model the coupling effects between variables like temperature, deformation, and degree of cure. An important contribution is that we have subsumed all modeling by using the free energy such that any possible restrictions of the simulation is recovered by revisiting the free energy definition. The computational implementation has been utilized by using an automatized generation of relations with the aid of symbolic differentiation of the assumed free energy. Therefore, any more advanced formulation is easily implemented into the framework developed herein. For demonstrating the strength of the numerical implementation as well as comprehending the material model’s capabilities, three different simulations have been utilized:

1. Two evolution equations, model A and B, are compared by means of the released heat in order to present the kink in the evolution denoting the glass transition temperature.
2. A realistic scenario has been used for demonstrating the vitrification in the case of an uncontrolled ambient temperature like the daily alternating temperature in Brussels.
3. Superposed hardening and mechanical loading is simulated during the cross-linking related glass transition change leading to a softening. This experimentally observed interplay between hardening and softening because of thermal and chemical contribution is possible by the proposed multiphysics simulation by adding glass transition dependence to the Young’s modulus. Additionally, the role of the heat convection across the boundaries has been discussed.

We stress that the code is publicly available in [73] for encouraging its use under GNU Public licensing [74].

## Footnotes

1 Taken from https://www.wunderground.com/dashboard/pws/IBRUSS7/graph/2019-10-1/2019-10-1/daily.
